# Peroxidase Activity of Human Hemoproteins: Keeping the Fire under Control

**DOI:** 10.3390/molecules23102561

**Published:** 2018-10-08

**Authors:** Irina I. Vlasova

**Affiliations:** 1 Federal Research and Clinical Center of Physical-Chemical Medicine, Department of Biophysics, Malaya Pirogovskaya, 1a, Moscow 119435, Russia; irina.vlasova@yahoo.com; Tel./Fax: +7-985-771-1657; 2 Institute for Regenerative Medicine, Laboratory of Navigational Redox Lipidomics, Sechenov University, 8-2 Trubetskaya St., Moscow 119991, Russia

**Keywords:** peroxidase activity, halogenating activity, reduction potential, myeloperoxidase, hemoglobin, cyt *c*/cardiolipin complexes, cytoglobin

## Abstract

The heme in the active center of peroxidases reacts with hydrogen peroxide to form highly reactive intermediates, which then oxidize simple substances called peroxidase substrates. Human peroxidases can be divided into two groups: (1) True peroxidases are enzymes whose main function is to generate free radicals in the peroxidase cycle and (pseudo)hypohalous acids in the halogenation cycle. The major true peroxidases are myeloperoxidase, eosinophil peroxidase and lactoperoxidase. (2) Pseudo-peroxidases perform various important functions in the body, but under the influence of external conditions they can display peroxidase-like activity. As oxidative intermediates, these peroxidases produce not only active heme compounds, but also protein-based tyrosyl radicals. Hemoglobin, myoglobin, cytochrome *c*/cardiolipin complexes and cytoglobin are considered as pseudo-peroxidases. Рeroxidases play an important role in innate immunity and in a number of physiologically important processes like apoptosis and cell signaling. Unfavorable excessive peroxidase activity is implicated in oxidative damage of cells and tissues, thereby initiating the variety of human diseases. Hence, regulation of peroxidase activity is of considerable importance. Since peroxidases differ in structure, properties and location, the mechanisms controlling peroxidase activity and the biological effects of peroxidase products are specific for each hemoprotein. This review summarizes the knowledge about the properties, activities, regulations and biological effects of true and pseudo-peroxidases in order to better understand the mechanisms underlying beneficial and adverse effects of this class of enzymes.

## 1. Introduction

Reactive oxygen species (ROS) are involved in many physiological processes, including signal transduction, cell proliferation, gene expression, angiogenesis, and aging. However, excessive ROS production initiates oxidative stress, which has been implicated in pathophysiological processes like diabetes, cardiovascular and neurodegenerative diseases, pulmonary diseases, sepsis and cancer [1,2]. As such, a variety of special mechanisms exist in the body for controlling the concentration and activity of molecules capable of generating oxidants and ROS.

The NADPH oxidase family of enzymes is a major source of ROS in vivo. The enzymes generate superoxide radicals (O_2_^•–^) that are the precursor of many ROS [1,3]. O_2_^•–^ can be spontaneously or catalytically (by superoxide dismutase) converted into hydrogen peroxide (H_2_O_2_). The major sources of O_2_^•–^ in plasma are NADPH oxidases on the surface of phagocytes and endothelial cells, xanthine oxidase bound to endothelial cells, and leakage from the mitochondrial respiratory chain [4,5,6]. A healthy physiological level of H_2_O_2_ in the plasma is 1–5 µM, but may be as high as 50 µM in inflammatory disease [6,7,8].

Hydrogen peroxide is one of the major in vivo oxidants. It is a two-electron acceptor with reduction potential *E_o_* (H_2_O_2_/H_2_O) of 1.32 V. However, H_2_O_2_ reacts poorly or not at all with most biological molecules due the high activation energy barrier that must be overcome to release H_2_O_2_ oxidative power [9] (see Supplementary Materials, Section S1).

The development of oxidative stress is mediated by transition metal ions, primarily with iron ions that promote H_2_O_2_ tunneling through the activation energy barrier. Various forms of iron react with hydrogen peroxide to form ROS. Table 1 lists the reaction rate constants of various forms of iron with H_2_O_2_ and the reduction potentials of radicals and oxidants formed in the reactions. The rate constants of the reaction of H_2_O_2_ with free iron or free heme are low. Ligation usually stimulates Fenton reactions. Notably, iron ions are strongly controlled in vivo. A system of ceruloplasmin-transferrin is responsible for the transport of free iron ions in plasma; intracellular iron redox activity is arrested by ferritin [10]. Iron chaperons guide iron delivery in cells directly to their “protein clients” thus limiting non-enzymatic redox-cycling reactions [11]. In plasma, a special protein hemopexin (Hx) binds free heme and inhibits its peroxidase activity [12].

Another group of molecules that interact with H_2_O_2_ are peroxidases. Peroxidases consume H_2_O_2_ and catalyze the one- or two-electron oxidation of a diverse array of Compounds called peroxidase substrates [1].

Heme-peroxidases are enzymes with a heme prosthetic group in the catalytic site where the iron is bound by four coordination bonds to a protoporphyrin IX derivative [28]. The rate constants of the interaction of heme in the active center of peroxidases with H_2_O_2_ are several orders of magnitude higher than that of the Fenton reaction [21] (Table 1). In the presence of H_2_O_2_, heme-peroxidases are activated to highly reactive intermediates. Lipid hydroperoxides also react with the peroxidase catalytic site. Except for cytochrome *c* (cyt *c*)/cardiolipin (CL) complexes [27], the rate of their reaction with heme is lower than that of H_2_O_2_ [29,30,31].

The mandatory requirements for the peroxidase activity of a heme-containing protein (i.e., its ability to form reactive intermediates) are:

(1) The peroxidases operate having iron in ferric form (Fe(3+) or Fe(III)).
(2) The heme iron of a peroxidase has five coordination bonds: four with nitrogens of tetrapyrrole ring, and the fifth heme ligand on the proximal heme side is a highly conserved imidazole ring of histidine residue linking the heme to the protein. On the distal heme side, the iron binds a water molecule. This H_2_O molecule is replaced by H_2_O_2_ upon activation of a peroxidase [28,32].
(3) The peroxidases are characterized by the specific location of amino acids in the active center for effective coordination and use of H_2_O_2_. On the distal heme site, a conserved histidine-arginine couple is involved in a hydrogen peroxide network [28,33].

**Figure 1:**
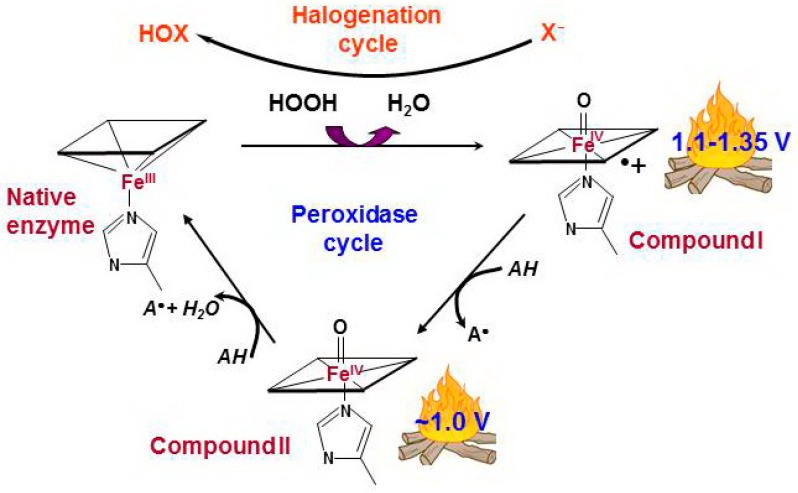
Peroxidase and halogenation cycles of human true peroxidases [34,35,36]. The interaction between hydrogen peroxide and native ferric peroxidase heme leads to the reduction of H_2_O_2_ to water with formation of Compound I which is oxoferryl porphyrin-π-cationic radical. Compound I can be sequentially reduced to the native enzyme via formation of Compound II by two one-electron oxidations caused by a number of simple compounds, peroxidase substrates (peroxidase cycle). AH is a peroxidase substrate that is oxidized with formation of a radical (A^•^). Compound I can catalyze the two-electron oxidation of (pseudo)halides, thus completing the so-called halogenation cycle. X^−^ stands for halide ions (Cl^−^, Br^−^, I^−^) and thiocyanate ions (SCN^−^). HOX is the corresponding (pseudo)hypohalous acid.

If a catalytic center of a hemoprotein meets the requirements listed above, H_2_O_2_ is specifically positioned in the peroxidase catalytic center and transfers its high oxidation potential to the heme. Two electrons are transferred from the enzyme to H_2_O_2_, which is reduced into water, whereby the heme is oxidized to Compound I (Figure 1). Compound I has two oxidizing equivalents: one in the oxyferryl heme center and the second is spread over the porphyrin ring as a porphyrin π-cation radical.

Compound I has very high oxidizing ability, with a reduction potential above 1.1 V, and it can oxidize many simple substances—the peroxidase substrates—through a one-electron mechanism via the intermediate Compound II—PorFe(4+)=O. At the end of the reaction, the active center returns to the native form, completing the peroxidase cycle.

Participation in the metabolism of nitric oxide and its derivatives is an important function of hemoproteins, including those that have peroxidase activity. This topic is discussed in a number of papers and reviews [37,38,39,40].

This review summarizes the author’s experience in working with different hemoproteins possessing peroxidase activity. Under inflammation or cellular disorders, relatively high levels of H_2_O_2_ can be generated, fueling peroxidase activity. Regulable production of oxidants and ROS is important for immune defense or as meaningful signals, but if out of control, can lead to the development of oxidative stress. Oxidative stress is implicated in the initiation and development of various pathologies. Antioxidants (glutathione, ascorbate, uric acid, etc.) are the first line of defense against radical species and oxidants. However, under oxidative stress, antioxidants are rapidly depleted, and then specific mechanisms to reduce oxidant production, including mechanisms for the inhibition of peroxidase activity, become significant. Due to differences in the structure and functions of peroxidases, the processes through which protein activities are limited differ. A variety of mechanisms protecting our body from excessive peroxidase activity prevent the transition of local disorders into systemic diseases.

## 2. Diversity of Human Hemoproteins with Peroxidase Activity

A wide variety of peroxidases exist, but most of them are plant, fungi, or bacteria peroxidases: horseradish peroxidase, ascorbate peroxidase, yeast cytochrome c peroxidase, lignin peroxidase etc. [41,42,43]. In the human body, fewer proteins display significant peroxidase activity. These enzymes can be divided into two groups: enzymes that are peroxidases by nature (true peroxidases), and enzymes that are converted into peroxidase under the influence of external conditions (pseudo-peroxidases) [21,25,26].

### 2.1. True Peroxidases 

True peroxidases are enzymes whose main function is to the generate oxidants and ROS. Myeloperoxidase (MPO), eosinophyl peroxidase (EPO), lactoperoxidase (LPO), and thyroid peroxidase (TPO) are homologous members of the mammalian peroxidase family. With the exception of TPO, the major physiological function of these enzymes is to contribute to innate immunity [21].

Compound I of the true peroxidases can be reduced by halide ions (Cl^−^, Br^−^, I^−^) or by thiocyanate (SCN^−^) via a two-electron mechanism (Figure 1). Reduction potentials of in vivo relative oxidants produced by peroxidases are presented in Table 1 (Supplementary Materials, Section S1). The capacity of human peroxidases to generate cytotoxic hypohalous (HOX, X = Cl^−^, Br^−^, and I^−^) and hypothiocyanous (HOSCN) acids accounts for their antimicrobial activity [33,44,45].

Although plasma concentration of SCN^−^ is equal to that of Br^−^ and 1000-fold lower than Cl^−^ (20–120 µM, 20–100 µM, and 100–140 mM, respectively), the relative abundance of SCN^−^ in biological fluids and its better electron donor capacity make it one of the main substrate for Compound I of peroxidases [22,33]. HOCl and HOBr are extremely powerful oxidants that react with biomolecules at diffusion-controlled rates. As any powerful oxidant (reduction potential >0.9 V; Table 1), they diffuse only a short distance and nonspecifically react with different biomolecules, causing considerable damage within a small radius of their production site [25]. Due to its lower reduction potential, HOSCN is a relatively long-lived oxidant that reacts specifically with SH groups. It can transfer oxidative potential of a peroxidase Compound I for longer distances with less harm to host tissues [46]. Bacteria proteins are sensitive to SH group oxidation [47].

Due to the low iodide concentration in vivo (<1 µM) with the exception of the thyroid gland, iodide oxidation by peroxidases is of little importance [33,36].

MPO, EPO and LPO are specifically structured for the synthesis of strong oxidants. Their active center is composed so that the protein moiety is not substantially damaged by local fire in the form of Compounds (Figure 1) and its oxidizing ability is directed toward the oxidation of substrates:

(1) The heme of the peroxidases is attached to protein moiety by at least two covalent bonds. The covalent linkages stabilize the position of the heme moiety, which is important for improving the redox ability of the heme and for the proper structural architecture of the substrate binding site [48].
(2) True peroxidases do not have oxidizable amino acids in close proximity to heme that are able to compete with external substrates for Compound I (except for cyclooxygenase).

The activity of the enzyme toward guaiacol, a hardly oxidizable phenolic compound, can serve as a test for the participation of peroxidase Compounds but not protein-derived radicals in substrate oxidation [49].

The substrate binding site of peroxidases is connected to the surface through a hydrophobic channel [28,50]. These enzymes prefer small anionic molecules as electron donors, such as halides, thiocyanate and small peroxidase substrates. Though the main function of true human peroxidases is the production of hypohalous acids and HOSCN, these enzymes can oxidize peroxidase substrates at neutral pH in the plasma. Numerous small molecular substrates, including phenolic compounds, aromatic amino acids, urate, ascorbate, nitrite, nitric oxide, serotonin, and others, are oxidized in the peroxidase cycle into radicals (Table S1) [51,52,53,54,55,56]. The oxidation of nitrite to nitrogen dioxide (^●^NO_2_) and of tyrosine to tyrosyl radical (Tyr-O^●^) forms potent oxidants that can participate in immune defense and simultaneously contribute to damaging host tissues (Table 1) [22].

The other two members of the true peroxidase family, namely cyclooxygenase (COX) and peroxidasins, are characterized by a multidomain structure and highly targeted catalytic activity. The major function of these enzymes is the oxidation of specific molecules.

COX (or prostaglandin H synthase) plays a key role in controlling the biosynthesis of various physiologically important prostaglandins. Contrary to other true peroxidases, the protein-derived (tyrosyl) radical is formed in the peroxidase cycle of COX. As a part of the COX catalytic cycle, this active radical is closely controlled and cannot oxidize external peroxidase substrates. Its redox activity is directed toward the oxidation of arachidonic acid, whose binding site is situated close to the tyrosyl radical [57].

The recently identified members of animal heme peroxidase family are peroxidasins (peroxidasin 1 and peroxidasin 2). Peroxidasin 1 is secreted to extracellular matrix and catalyzes the oxidation of bromide to hypobromous acid. HOBr mediates the formation of specific sulfilimine cross-links of collagen IV in the basement membrane, which is important for the structural integrity of extracellular matrix [58,59]. As with TPO, COX and peroxidasins generate oxidants to perform biosynthesis tasks. These enzymes cannot produce excess of highly active oxidants and ROS and initiate oxidative stress.

### 2.2. Pseudo-Peroxidases

Pseudo-peroxidases are hemoproteins that perform different important functions in the body and initially are not meant to interact with H_2_O_2_. However, due to changes in external conditions, the characteristics of their active center can change, and they can display peroxidase-like activity. The pseudo-peroxidase active sites are not specifically designed to catalyze the H_2_O_2_ reduction to water. The rate of reaction of heme with H_2_O_2_ is rather low, so the first step of the pseudo-peroxidase reaction is rate-limiting (Table 1). Therewith, hydroxyl radicals can be formed in the active center due to homolytic splitting of H_2_O_2_ [60,61]. Nevertheless, these proteins consume H_2_O_2_ and oxidize peroxidase substrates, so they may be considered as peroxidases.

**Figure 2:**
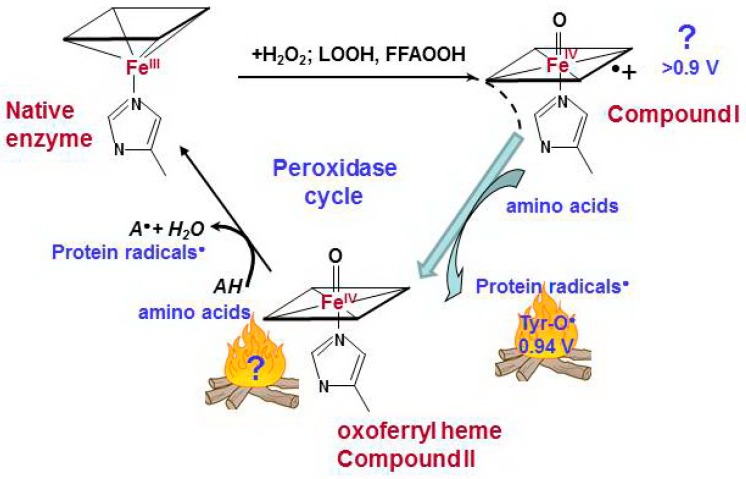
Catalytic cycle of pseudo-peroxidases [40,63,68,69]. The interaction between hydrogen peroxide and native ferric pseudo-peroxidase heme leads the formation of Compound I which is most likely oxoferryl porphyrin-π-cationic radical. Compound I immediately oxidizes amino acid residues (Tyr, Trp, His) which are located near the heme with formation of protein based radicals and oxoferryl heme. Oxoferryl heme iron can oxidize protein amino acids and peroxidase substrates. AH is a peroxidase substrate that is oxidized with formation of a radical (A^•^). The protein-based tyrosyl radicals are the alternative reactive intermediates of pseudo-peroxidases.

Pseudo-peroxidases are different in terms of their heme and activity characteristics, but all of them have one common feature: exposure to H_2_O_2_ causes immediate oxidation of protein amino acids that are close to the active center (most commonly tyrosine, tryptophan, or histidine) [62,63]. Compound I was not detected for pseudo-peroxidases. The reaction of heme with H_2_O_2_ results in formation of oxoferryl heme PorFe(IV)=O, equivalent to Compound II of true peroxidases, and protein-derived radicals (Figure 2). The protein-based tyrosyl radicals are the alternative reactive intermediates for the oxidation of larger substrates that cannot access the heme pocket [64]. Hydroxyl radicals, which can be formed in the pseudo-peroxidase active site due to the homolytic splitting of H_2_O_2_, are unlikely responsible for tyrosyl radical formation. First, hydroxyl radicals are non-specific oxidants. Secondly, phenoxyl radicals are the minor products of the reaction of HO• with phenolic compounds (<5%) (Figure S1) [65,66,67].

Pseudo-peroxidases can be divided into two groups:

(1) Proteins in which the heme iron initially does not have an amino acid ligand in a distal coordination position—hemoglobin (Hb) and myoglobin (Mb).
(2) Proteins in which the heme iron has a sixth coordination bond, but under the influence of external factors, the configuration of the active site can change, and this newly conferred peroxidase activity plays an important role in the cells, unrelated to the primary biological functions of the enzymes.

Cyt *c* and cytoglobin (Cygb) are hexacoordinated proteins with methionine/histidine residues occupying the sixth coordination sites of the heme iron (for Cygb—under reducing conditions). Anionic lipids can break this bond and awaken the dormant peroxidase activity of cyt *c* and Cygb [26,70,71,72].

Low temperature electron paramagnetic resonance (LT EPR) spectroscopy is a powerful tool used to study peroxidase activity of hemoproteins (Figure S2). EPR signal at a g-factor of about six (g ~ 6) evidences the absence of a ligand at the sixth coordination position of iron. Protein oxidation and the formation of protein-derived (tyrosyl) radicals can be monitored by acquiring the spectra of tyrosyl radicals at a g-factor of about two (g ~ 2). Not only H_2_O_2_, but other small molecules including CO and NO, can interact with iron at the sixth coordination position side. NO binding to both heme-Fe(III) and heme-Fe(II) changes the optical absorbance spectra of hemeproteins in the Soret band region, but only Fe(II)-NO complexes have characteristic LT EPR spectra of nitrosylated heme (Figure S2). The LT EPR method has been successfully employed for the study of pseudo-peroxidases and provided unique useful information [64,72,73,74,75,76,77].

## 3. Myeloperoxidase

MPO is the queen of the peroxidase family. It is the only enzyme capable of generating physiologically significant amounts of HOCl—the potent oxidizing agent that is necessary to fight invading pathogens [78,79].

The enzyme is stored in the azurophilic granules of neutrophils, comprising approximately 5% of cellular dry weight. During neutrophil activation, the granules release their contents, including MPO, into the phagosomes and the extracellular space. Simultaneously, NADPH oxidase assembles on the plasma membrane and generates superoxide radicals that dismutate to form H_2_O_2_ [1,78]. In inflamed tissue, steady state concentrations of H_2_O_2_ in extracellular medium can be as high as 100 µM [6,8]. Upon phagocytizing a microbe, MPO is released into the phagosome up to a concentration of about 1 mM [35]. MPO is one of the key components of neutrophil extracellular traps (NETs). NETs are formed during neutrophil-specific cell death, characterized by the release of DNA strands associated with histones and decorated with about 20 different proteins [80,81].

Under physiological conditions, MPO Compound I oxidizes similar amounts of SCN^−^ and Cl^−^ [82]. Nevertheless, a major function of neutrophil myeloperoxidase is the synthesis of HOCl, which plays a cytotoxic role against bacteria and viruses at inflammatory sites and in phagosomes [79].

The concentration of MPO in plasma is normally in the range of 20–100 ng/mL, whereas under pathology, these values increase several-fold [83,84,85]. Despite of the low concentration of MPO in vivo, the enzyme has been implicated in the development of a number of widespread diseases [21], such as cardiovascular disorders [83,86,87,88], Alzheimer’s disease [89,90], kidney diseases [91,92], and rheumatoid arthritis [93].

The enzyme is a 145–160 kD homodimer comprising two identical subunits joined by a single disulfide bridge. Each subunit is a complex of a light chain and a heavy chain; the latter contains heme. In addition to the two ester linkages that are also present in EPO and LPO, the heme in MPO has a unique sulfonium ion linkage that significantly distorts the prosthetic group from a planar conformation [48,94,95]. As a result, myeloperoxidase Compound I is the strongest one- and two-electron oxidant among all peroxidase compounds. The reduction potentials are 1.35 and 1.16 V for the Compound I/Compound II and Compound I/native MPO couples, respectively [22,96].

**Figure 3:**
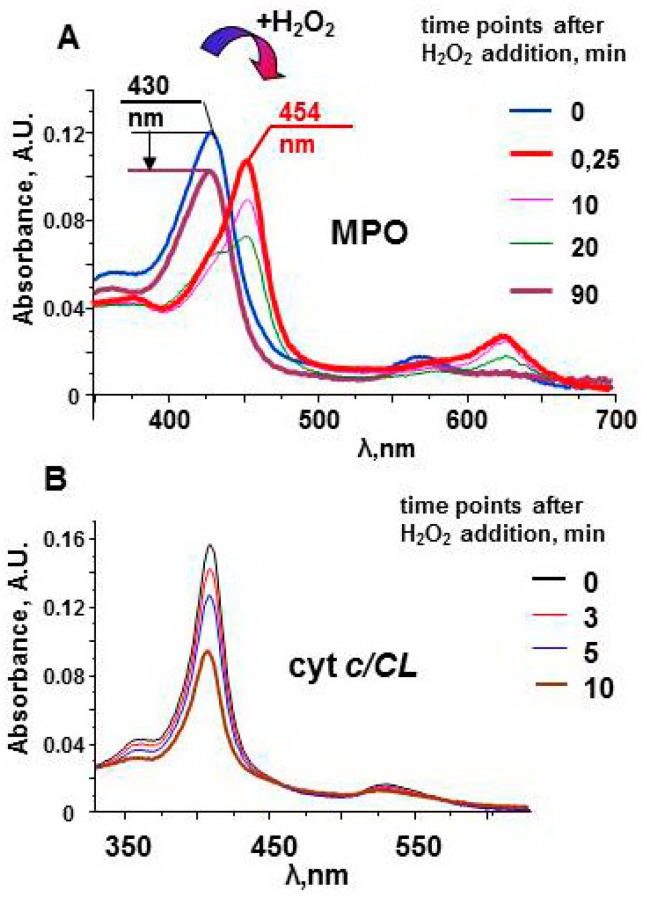
Optical spectra of myeloperoxidase and cyt *c*/TOCL complexes after addition of H_2_O_2_ [51,97]. (**A**) Upon addition of an excess of H_2_O_2_ (200 μM), MPO (1.2 μM) undergoes conversion from its native form (the Soret band maximum at 430 nm) to Compound II having the absorbance maximum at 454 nm. In the absence of peroxidase substrates the absorbance at 454 nm slowly decreases, and the peak shifts towards 430 nm. The arrow indicates a decrease in the Soret band intensity in 90 min after addition of H_2_O_2_ to MPO. (**B**) A progressive decrease of the absorbance of cyt *c*/TOCL during its auto-oxidation process initiated by the addition of 250 μM H_2_O_2_ to the solution of ferri-cyt *c* (1.5 μM, tetraoleoyl-CL 30 µM). 50 mM sodium phosphate buffer, 100 mM NaCl, pH 7.4, 100 µM DTPA.

The protein does not have oxidizable amino acids near the active site. In the absence of external substrates, MPO compounds are long-lived oxidants. The stability of Compound II can be observed by measuring the protein spectra in the Soret band region (Figure 3). The spectrum of the native enzyme has a maximum at 430 nm. Interaction of MPO heme with H_2_O_2_ leads to the immediate formation of Compound I. The one-electron oxidation of Compound I by the next molecule of H_2_O_2_ results in formation of Compound II and superoxide [54]. Compound II has characteristic spectrum with maximum at 454 nm. Compound II is stable and can wait for a substrate for a relatively long time [51]. Only after ~90 min, the enzyme returns to the native form. The decrease in the magnitude of the spectrum evidences partial damage of MPO. However, protein-derived radicals cannot be detected by LT EPR spectroscopy after the reaction of MPO with hydrogen peroxide.

Although the major function of peroxidases in innate immunity is the production of hypohalous acids and HOSCN, these enzymes can also oxidize peroxidase substrates under plasma conditions (neutral pH and 140 mM NaCl; Table S1) [51,53]. Amino acid residues of proteins, such as tyrosine and tryptophan, are peroxidase substrates [98,99]. Does this mean that MPO can directly oxidize proteins in plasma? There are several mechanisms for controlling the ‘fire’ in the MPO active site to minimize its adverse impact on biomolecules.

### 3.1. Restricted Access to MPO Active Site 

The structure of the MPO active site prevents the enzyme from oxidizing large molecules. The active site of MPO is located at the base of a deep and narrow heme pocket inaccessible to compounds significantly larger than a dipeptide [100,101]. For macromolecule oxidation, small substrates are needed, which are the intermediates between Compounds and the biomolecules. At pH < 7, the major oxidant is HOCl. At neutral pH, free radicals with high reduction potential formed in the peroxidase cycle, mainly phenoxyl radicals and nitrogen dioxide (Tyr-O^●^, ^●^NO_2_), can mediate oxidation of plasma constituents (Table 1).

The accessibility of the MPO active site can be tested by substrates with different characteristics. Table 2 presents the activity of different hemoproteins toward phenolic compounds that are peroxidase substrates, namely guaiacol, Amplex red and etoposide. The substrates differ in molecular mass and reduction potentials (Figure S3). Guaiacol oxidation with formation of tetraquaiacol can be observed only by peroxidase Compounds. MPO activity toward this indicative substrate is about 1000-fold higher than for other proteins. Two other tested substrates—Amplex Red and etoposide—have lower reduction potentials compared to quaiacol. So, they are expected to be oxidized more easily. However, MPO activity toward Amplex red is more than 10 times lower, and that for etoposide is 3 × 10^4^ times lower than activity toward guaiacol. Despite its low reduction potential (*Eo* = ~0.56 V), etoposide is a very poor substrate for MPO because access of this large phenolic compound (MW = 589 D) to the MPO active site is highly constrained [102,103].

The small phenolic molecules may act as co-substrates for oxidation of large and bulky compounds, such as etoposide, because they have free access to the MPO active site and due to the relatively high oxidizing potential of their phenoxyl radicals compared with etoposide (Table 2, compare MPO and MPO + phenol) [103]. In this case, nonspecifically reactive phenoxyl radicals, which oxidize different biomolecules, are converted to etoposide-O^•^, which reacts specifically with GSH and protein SH groups. As the etoposide phenoxyl radical is relatively long-lived compared with the phenol radical [102], the oxidative potential of MPO can be more readily transferred. The activity of many proteins is sensitive to the redox state of thiols [105]. The re-oxidation of phenolic compounds results in the decrease in MPO oxidative ability (reduction potentials: Compound I > tyrosyl-O^•^ > etoposide-O^•^). Tyrosine is the only phenolic compound that is present in plasma in free form (Table S1). Other phenolic compounds can enter the body as medicines or with food. These phenolic compounds may be antioxidants (flavonoids) or may have a weak oxidative activity, thereby altering the profile of MPO-induced damage to biomolecules [106,107,108,109].

### 3.2. Ceruloplasmin Is an Endogenous MPO Inhibitor

The interaction of highly cationic MPO (pI ~ 10) with anionic proteins and with the negatively charged surfaces of different cells has been suggested to be mostly dependent on electrostatic interactions [110]. The weak binding of MPO to albumin and binding to lipoproteins have been demonstrated [4,84,111]. Several negatively charged proteins are present in plasma at micromolar concentrations, like ceruloplasmin (CP), fibrinogen, and haptoglobin. Immunoprecipitation experiments revealed that CP is the major protein in plasma that is associated with MPO [112,113].

CP is a globular anionic copper containing plasma glycoprotein (pI ~ 4.4, MW ~ 132 kD) that has multiple functions, the main one of which is ferroxidase activity, i.e., oxidation of redox-active Fe^2+^ to Fe^3+^. Trapping of Fe^3+^ by transferrin prevents iron participation in redox chemistry [114]. CP is an abundant acute phase protein whose concentration can reach the value of 4–10 µM with pathologies [115,116].

CP binds to MPO with stoichiometry 2:1: one molecule of CP binds to each monomer of MPO with a dissociation constant of ~0.15 µМ [117]. Binding of CP in close proximity to the MPO active center results in inhibition of both the halogenating and peroxidase activities of MPO [118,119,120,121]. The degree of inhibition depends on the size of the peroxidase substrate, suggesting that CP hinders access of larger substrates to the MPO active site [121,122]. Reduction of Compound I to Compound II and then retardation of the turnover of Compound II to native enzyme is the mechanism of CP-induced inhibition of MPO activity [113]. Oxidation of ascorbate by MPO was observed in plasma from CP-knock-out mice, but no significant loss of the antioxidant in plasma from wild type animals was detected [113]. Partially proteolized or thrombin-damaged CP loses the ability to inhibit MPO. Therefore, thrombin can exacerbate inflammation and development of pathologies by impairing MPO inhibitory function of CP [123].

### 3.3 pH-Dependent Rregulation of MPO Activity

The chlorinating activity of MPO decreases with increasing pH [124,125]. The rate constant of the reaction of Cl- with Compound I is about 150-fold higher at pH 5.0 compared to pH 7.0, but this does not imply a decrease in the oxidative capacity of MPO. At acidic pH, MPO catalyzes the formation of HOCl, whereas oxidation of phenolic substrates in the peroxidase cycle can be observed at neutral pH [104,126]. The ratio between peroxidase and chlorinating MPO activities is important for targeted synthesis of HOCl at inflammatory sites and in phagosomes, where pH may be shifted into the acidic range [127].

Peroxidase substrates can dose-dependently inhibit the formation of HOCl at neutral pH [104,128,129]. The substrates of the MPO peroxidase cycle are present in plasma and the intracellular space in low concentrations, but their diversity suggests that they may compete with halide ions during interaction with Compound I (Table S1).

## 4. Eosinophil Peroxidase and Lactoperoxidase

The standard reduction potential of the redox couple Compound I/ferric enzyme of human true peroxidases falls in the order of MPO (1.16 V) > EPO (1.10 V) > LPO (1.09 V), which is proportional to the reduction potential of oxidants the peroxidases can produce in the halogenation cycle (Table 1) [22,96,130]. The difference in the oxidative ability of the enzymes is due to the variations in the active site structure and properties [28,50].

Eosinophil peroxidase is the major granule enzyme of eosinophils, which are the phagocytic cells recruited against invading bacteria, viruses, protozoans and helminths [45]. EPO is a monomer comprised of light and heavy chains (57 and 11 kD, respectively). Heme is bound by one ester bond to the protein, whereas a second ester linkage is formed autocatalytically in the presence of hydrogen peroxide [131]. At normal plasma conditions, the enzyme catalyzes oxidation of bromide and thiocyanate to HOBr and HSCN, respectively. The enzyme has limited ability to generate HOCl at acidic pH, so the EPO-induced chloride oxidation in vivo is meaningless [132]. EPO plays a prominent deleterious role in pathogenesis of various common human allergic disorders, including asthma [133,134,135].

More than 60% of amino acids residues are identical in EPO and MPO, and even higher homology was demonstrated for the active-site-related residues [136]. Expectedly, the mechanisms of protection against undesirable redox activity of EPO are similar to those described above for MPO. If the pH of the medium changes from 7 to 5, the brominating activity increases by an order of magnitude (from 10^−7^ to 10^−8^ M^−1^ s^−1^) [132]. The active center of EPO is inaccessible to plasma macromolecules, similar to MPO [50]. EPO forms a complex with CP, but the stoichiometry of binding is 1:1. CP inhibits the peroxidase activity of EPO but does not affect the brominating activity of the enzyme [137].

Lactoperoxidase was found in mucosal surfaces and exocrine secretions including milk, tears, and saliva, and in nasal and lung airway fluids. LPO is a major contributor to airway defenses and to the antimicrobial properties of exocrine gland secretions [33,36,138,139].

LPO is monomeric hemoprotein (MW ~ 78 kD). The prosthetic group is bound to the protein autocatalytically; noncovalently bound heme becomes covalently bound through two ester linkages after enzyme exposure to H_2_O_2_ [140]. At physiological conditions, LPO has barely detectable activity with bromide but oxidizes iodide and thiocyanate. SCN^−^ is the physiological substrate of LPO because it is much more abundant than iodide [33,141,142] Normal plasma levels of thiocyanate are 20–120 µM (iodide < 1 µM). Thiocyanate and iodide are actively accumulated in secretory epithelial cells through uptake from blood by the sodium–iodide symporter. The maximum concentration of thiocyanate was observed in saliva (2–4 mM), and that of iodide was in milk (2 µM) [33,36]. HOSCN formed by LPO is a weak oxidizing agent that reacts primarily with thiols and inhibits microbial metabolism and growth [47,142].

The substrate channel of LPO is longer, narrower, and more hydrophobic compared to that of MPO, which prevents direct oxidation of macromolecules by the enzyme [36,50].

Thyroid peroxidase is localized in membranes of thyroid follicle cells. The enzyme generates HOI and is involved in the synthesis of the thyroid gland hormones: thyroxine and triiodothyronine [21].

## 5. Hemoglobin and Myoglobin

The main function of respiratory hemoproteins, such as Hb and Mb, is the storage and delivery of oxygen into cells. Both proteins are members of the globin family, which also includes Cygb and neuroglobin. Mammalian globins have a typical structure: a protein consists of six to eight helical chains arranged in a three-on-three helix ‘‘sandwich’’, forming the hydrophobic pocket of the active center containing heme [25]. All members of the globin family possess NO dioxygenase activity and participate in NO metabolism [143,144,145].

**Figure 4:**
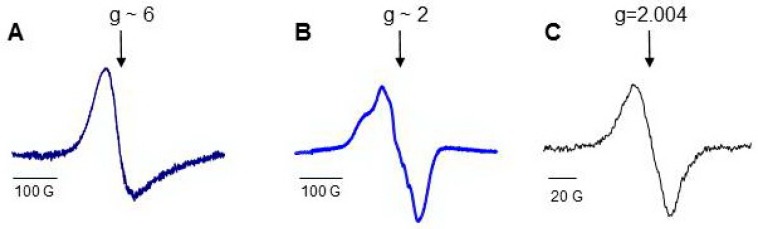
Low-temperature EPR (77 K) spectra of Hb and Mb [69]: (**A**) low field EPR signal of ferric myoglobin; (**B**) a typical LT EPR signal of heme-nitrosylated Hb(II); (**C**) spectrum of protein-derived (tyrosyl) radicals measured in 30 s after addition of H_2_O_2_ to Hb.

Hb consists of four identical subunits (MW ~ 64 kD), and Mb is a monoglobular protein (MW ~ 16.7 kD). Proteins perform their functions, having iron in the active center in the ferrous (Fe(II)) state with free sixth coordination bond. The characteristic EPR signal of open heme measured at g ~6 can be detected from the proteins at the temperature of liquid nitrogen (77 K), and heme can be easily nitrosylated (Figure 4) [69,146]. There are oxidizable amino acids in close proximity to heme; the addition of H_2_O_2_ to protein solutions causes formation of protein-derived radicals [64,147].

The redox activity of Hb and Mb is suppressed in myocytes and erythrocytes, respectively, by the reducing environment of the cells. The ferric form of Hb comprises about 1% of total Hb in erythrocytes. Ferric Hb is effectively converted into ferrous protein by metHb reductase [148]. Myocyte injury or hemolysis cause hemoprotein leakage into tissues and blood where the globins do not have their normal reducing environment. Since the redox chemistry is the same for Hb and Mb, but the aggravation of a number of pathologies is more associated with hemolysis and the release of Hb into the plasma, this chapter mainly focuses on hemoglobin.

A constant process of mild intravascular erythrocyte damage occurs under normal conditions, so that free hemoglobin appears in plasma in small amounts. The inadvertently appearing ferric form of Hb can be converted into ferrous Hb by ascorbate. Given the lack of oxidizing equivalents, the protein cannot display any peroxidase activity.

Hemolysis occurs in many diseases such as trauma, sepsis, and sickle cell disease. Under some pathological conditions, particularly as a result of microangiopathic hemolytic anemia, concentrations of Hb in plasma can reach micromolar levels [149]. Under uncontrolled pathological conditions accompanied by inflammation and oxidative stress, depletion of antioxidants and generation of superoxide radicals by activated neutrophils induce the redox activity of the hemoproteins, resulting in damage to plasma proteins and lipids [62]. The globin-based free radicals of ferryl hemoglobin were detected in normal human blood upon H_2_O_2_ treatment [150].

Ferrous Hb is activated by H_2_O_2_ to the oxoferryl form, equivalent to Compound II. The second molecule of H_2_O_2_ can convert the active site into the ferric form. The ferric form of the protein is oxidized by H_2_O_2_ to Compound I, which, in the absence of exogenous peroxidase substrates, immediately oxidizes a tyrosine residue near the heme [77,151,152]. The peroxidase substrates can be oxidized in two processes: by Compounds, if heme in hydrophobic pocket is accessible for a substrate; or by a tyrosyl radical, which is exposed to the peroxidase surface (Figure 2) [64]. The relatively high oxidation ability of Hb and Mb toward guaiacol compared to cyt *c*/CL supports the participation of Compounds in substrate oxidation (Table 2). Mediation of a substrate oxidation by tyrosyl radicals accelerates heme reduction but decreases the oxidative potential of a peroxidase.

Self-oxidation of Hb and formation of protein-derived (tyrosyl) radicals result in protein cross-linking and aggregation. Tyrosyl radicals recombine with the formation of very stable carbon-carbon covalent bonds between the proteins. This process involves not only a hemoprotein itself but other proteins in plasma. As a result, large protein hetero-oligomers (aggregates) occur [69].

Reeder et al. demonstrated the formation of a covalent link between the heme and protein moiety for Hb or Mb treated with hydrogen peroxide [25,153]. Globins with covalently-linked heme showed higher peroxidase activity and cellular toxicity. Oxidation of low density lipoproteins by heme-to-protein cross-linked Mb was five times greater than that of native protein [154]. The heme-to-protein cross-linked Mb or Hb are stable forms of the proteins, and the authors identified them in urine, kidney, and cerebrospinal fluid [25].

Contrary to cyt *c* or Cygb, the peroxidase activity of Hb does not make any biological sense, so it should be neither regulated nor limited but just abrogated. There are two special acute phase proteins in plasma whose major function is to prevent adverse unintended consequences of Hb release [155].

### 5.1. Haptoglobin 

Haptoglobin (Hp) is an abundant plasma protein with high binding affinity toward Hb (Kd ~ 10^−15^ M, [156]), reminiscent of an antigen-antibody reaction. Human haptoglobin occurs as three major phenotypes: Hp 1-1, Hp 2-1, and Hp 2-2. The phenotypes differ in the protein structure and their distribution throughout different populations in different locations [157]. Nevertheless, all Hp types function to bind free Hb and to facilitate its delivery into macrophages via the CD163 receptor-mediated pathway [158,159,160,161]. Circulating haptoglobin provides an important endogenous defense against the toxic effects of Hb.

Hp is an acute phase protein; its normal plasma level ranges from 0.1 to 2 mg/mL, which increases two- to five-fold during inflammation [12,156]. These amounts of Hp are sufficient to bind micromolar levels of free Hb [69].

Trapping of free Hb by Hp results in the formation of Hb-Hp complexes. Hp protects Hb from heme dissociation, thus preserving the peroxidase activity of hemoprotein. Hb-Hp complex formation does not alter Hb’s ability to consume H_2_O_2_ [24]. In complex with Hp, the oxidizing potential of Hb is partially re-directed toward oxidation of Hp, thus delaying the oxidation of plasma constituents. Oxidation of Hb-Hp complexes causes covalent cross-linking of the proteins and the formation of large aggregates. The ratios of peroxidase activity were 100:50:10 for Hb, Hb-Hp complexes, and Hb-Hp aggregates, respectively. The aggregates still displayed significant peroxidase activity toward external molecules [69].

Hb-Hp aggregates were uptaken by macrophages at rates exceeding those for Hb-Hp complexes. The engulfed Hb-Hp aggregates induced intracellular oxidative stress and a dose-dependent cytotoxicity. Neither Hb nor Hb-Hp complexes showed significant toxic effects under the same incubation conditions [69]. Long-living protein-based (tyrosyl) radicals in Hb-Hp aggregates have high oxidizing potential and may be responsible for the cytotoxicity of Hb-Hp aggregates. Notably, the lifespan of protein-based radicals in aggregates (10–20 min) is comparable to the lifespan of Hb-Hp complexes in blood (<10 min) [12,69].

Hb-Hp aggregates were found in septic plasma [69]. In the hemolysis setting, Hb may intensify the potentially fatal effects of sepsis syndrome in patients with trauma, infection, or hypotension [162,163]. Mb, released from damaged muscle (rhabdomyolysis), cannot be bound by Hp, leading to kidney damage [62].

### 5.2. Hemopexin

Hx is a plasma protein that functions to sequestrate free heme or extract heme from other proteins and transport it into hepatocytes by receptor-mediated endocytosis [12]. The enzymatic degradation of heme by the heme oxygenase enzyme system leads to the formation of biliverdin, with the concomitant liberation of CO and reduced heme iron [164]. The human body generates more than 12 mL of CO per day from heme catabolism [165].

In adults, serum Hx concentration ranges between 0.4 and 1.5 g/L. In the acute phase, Hx concentration increases two- to three-fold [12,155]. When Hp is exhausted, free ferrous Hb can be oxidized in plasma to its ferric form. Non-covalently bound heme of Hb(III) can be easily uptaken by hemopexin (but not from ferrous Hb or Hb bound to Hp) [155,166].

When free Hb(III) is exposed to H_2_O_2_, self-oxidation of Hb causes conformational changes in the heme pocket environment, facilitating the release of heme from the protein. Free heme is a highly hydrophobic compound that cannot exist as a soluble molecule under neutral pH, heme is trapped immediately by albumin or lipoproteins, which have middle affinity for heme but can compete with Hx due to their high concentrations in plasma. Albumin has special sites for heme binding characterized by K_d_ ~2 × 10^−8^ M [167], whereas incorporation of heme into lipoproteins can initiate their oxidation and aggregation. Albumin-heme complexes have peroxidase activity and oxidize phenolic compounds [19,167], but this activity is unlikely relative to the in vivo situation. Having high binding capacity toward heme (K_d_ ~ 10^−13^ M), Hx sequesters heme from other proteins in a ratio of 1 mol heme per 1 mol Hx and inhibits peroxidase-like activity of heme by 80–90% [19,155]. Thus, despite the relatively long life time of the complex Hx-Hb in plasma (7–8 h, [12]), bound heme does not have significant adverse effects on cells and macromolecules.

## 6. Intracellular Pseudo-Peroxidases

### 6.1. Cytochrome c/Cardiolipin Complexes

Cyt *c* is a heme protein located in the intermembrane space of mitochondria where it shuttles electrons between complex III (ubiquinol:cytochrome c reductase) and complex IV (cytochrome c oxidase), participating in the life-supporting synthesis of ATP [168]. Another important function of cyt *c* is participation in programmed cell death through apoptosis. Cyt *c* is released from mitochondria into the cytosol and amplifies signals that are generated by other apoptotic pathways. Both electron transport function and propagation of apoptosis are well-accommodated by six-coordinated heme iron in the active center of the protein [169].

The heme of cyt *c* (MW ~ 12.5 kD) is linked by a covalent bond to the only SH group of the protein. The heme iron is coordinated by His18 at the proximal position and by Met80 on the distal side. Significant conformational changes accompanied by elimination of the distal heme ligand may be induced by strong denaturating agent such as guanidine chloride or by protein oxidation by hypochlorous acid [170,171]. The peroxidase activity of cyt *c* increases substantially after protein unfolding and oxidation.

Similar structural changes in cyt *c*, accompanied by the loss or exchange of Met80 and an increase in peroxidase activity, were observed when cyt *c* interacted with negatively charged phospholipid membranes [26,172,173,174,175]. The strength of phospholipids in inducing activation of cyt *c* into peroxidase ranks as follows: CL ≈ phosphatidic acid (PA) > phosphoinositols (PI) > phosphatidylserine (PS) >> phosphatidylcholine (PC) [70]. The interaction of cyt *c* with CL is of great importance because both molecules are components of the mitochondria interior. Millimolar levels of (ferri)cyt *c* are contained in the intermembrane space [176].

**Figure 5:**
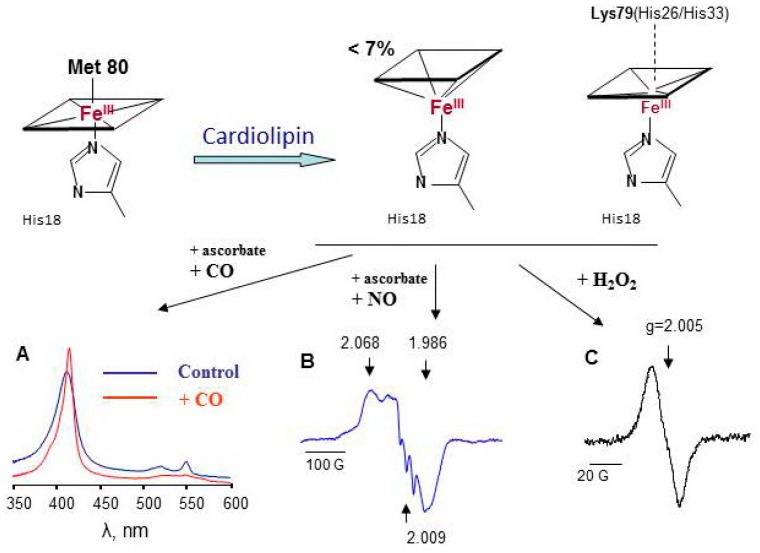
Cardiolipin induces protein unfolding of cyt *c* accompanied by the loss or exchange of the distal heme iron ligand resulting in an increased accessibility of heme for small molecules [26,75]. (**A**) absorbance spectra of native cyt *c*(II)/CL complexes and complexes incubated with CO; (**B**) LT EPR spectrum of nitrosylated cyt *c*(II)/CL complexes, (**C**) LT EPR spectrum of cyt *c*(III)/CL complexes exposed to H_2_O_2_ for 30 s.

Cardiolipin is a mitochondria-specific phospholipid that is located predominantly in the inner leaflet of the inner mitochondria membrane in normal cells. Tetralinoleoyl cardiolipin (TLCL) is specific for cardiac and skeletal muscles; in other cells, highly diversified forms of CL are present. In apoptotic cells, the CL content both in the outer leaflet of the inner membrane and in the outer mitochondrial membrane markedly increases [177]. As a consequence, the concentration of cyt *c*/TLCL complexes increases. CL causes partial unfolding of cyt *c* and weakening of the coordination bond between heme-iron and Met80. Contrary to Mb (Figure 4A), characteristic LT EPR signal at g ~ 6 could be hardy resolved under special experimental conditions in cyt *c*/CL EPR spectra at 77 K [26]. Less than 7% of the protein loses the distal heme ligand upon interaction with CL. Lys 79, His26, or His33 are the likely candidates for the substitution of the position of Met80 [178,179]. Because these amino acids are weak ligands of heme iron in cyt *c*/CL complexes, small molecules, like H_2_O_2_, NO, and CO, gain access to the heme site of the protein (Figure 5) [180,181]. The dependence of the magnitude and shape of cyt *c*-CO and cyt *c*-NO complex spectra on CL concentration shows that the increase in CL potentiates conformational changes of cyt *c* and enhances heme accessibility for small molecules (Figure S4) [75]. At relatively high CL concentrations (cyt *c*:CL > 1:15), a three-line signal with a splitting of 17 G emerges at g = 2.009 in the LT EPR spectra of nitrosylated cyt *c*/CL (Figure 5B). This three-line signal is due to the shift in electron density in Fe-N bond to nitrogen (nuclear spin I = 1) due to the cleavage of the His18-Fe(II) bond [40].

Exposure of cyt *c*/CL complexes to hydrogen peroxide results in immediate formation of protein-derived (tyrosyl) radicals (Figure 5C). For cyt *c*/CL, no evidence of Compound I or II was found; protein amino acids immediately donate electrons to porphyrin radical and probably to oxoferryl intermediate (Figure 2). In the absence of antioxidants or peroxidase substrates, the heme of cyt *c*/CL complexes degrades within several minutes after addition of H_2_O_2_ (Figure 3B). Among the four tyrosines of cyt *c*, Tyr67 is a likely electron donor for Compounds formed in the cyt *c* active site with further transfer of the radical reaction to other oxidizable amino acids [63]. As the oxidative ability of tyrosyl radicals is lower than that of peroxidase Compounds, cyt *c*/CL complexes display relatively lower peroxidase activity toward quaiacol compared to other hemoproteins, but their capacity to oxidize a lipid-soluble phenolic compound etoposide (*Eo* = 0.56 V, [102]) is the highest (Table 2). The active site of cyt *c*/CL is occupied by an acyl chain of CL. Lipids and lipid soluble compounds are better substrates for cyt *c*/CL peroxidase.

The peroxidase activity of cyt *c*/CL toward external peroxidase substrates is low; the major biological function of these complexes is oxidation of the polyunsaturated fatty acids (PUFA) of CL. Cyt *c*/CL peroxidase activity is implicated in the propagation of apoptosis. In mitochondria, the peroxidase activity of the cyt *c*/CL complex is specific toward oxidation of PUFA-CL, yielding FA-hydroperoxides. More abundant phospholipids, PC and PE, do not undergo peroxidation [26]. Cyt *c* dissociates from peroxidized CL; peroxidized CL participates in mitochondrial membrane permeabilization, resulting in the release of cyt *c* and other pro-apoptotic factors from mitochondria to cytosol [177]. The second important function of cyt *c*/CL peroxidase is the generation of a high diversity of polyunsaturated molecular species for lipid mediator production. Cyt *c*-oxydized CL undergoes phospholipase A_2_-catalysed hydrolysis thus generating multiple oxygenated fatty acids, including well-known lipid mediators [182].

To activate cyt *c* into peroxidase, H_2_O_2_, which is formed due to a leaking electron transport chain (from O_2_^•−^ dismutation), reacts with the protein active site. After several catalytic cycles, lipid hydroperoxides accumulated. Fatty acid hydroperoxides more effectively react with the cyt *c* active site compared to H_2_O_2_, so they can accelerate the CL peroxidation (Table 1) [27]. Accumulation of LOOH could heavily potentiate cyt *c*/CL peroxidase activity, promoting cell death. Implication of cyt *c* peroxidase activity in neurodegenerative disorders may be due to the cyt *c*/CL complex oxidation and formation of protein-lipid aggregates [183,184].

The mechanisms preventing elevation of cyt *c*/CL peroxidase activity in mitochondria are based on the restriction of H_2_O_2_ and substrate access to cyt *c* active site [40,75,177]:

(1) In resting-state mitochondria, most CLs and cyt *c* are spatially separated; therefore, the peroxidase activity of cyt *c* is not significant.
(2) The active center of cyt *c* is occupied by an acyl chain of CL, which prevents access of peroxidase substrates to Tyr67 in the active site.
(3) Tyrosyl radicals exposed on the cyt *c* surface can recombine with the formation of very stable dityrosine bonds. As a result, cyt *c*/CL complexes can form aggregates, and the effective concentration of cyt *c* that can react with H_2_O_2_ decreases.
(4) Mitochondrial NO, from external sources and generated by mitochondrial nitric-oxide synthase, plays a regulatory role in the control of peroxidase activity of cyt *c*/CL complexes.

Design and synthesis of mitochondria-targeted inhibitors of cyt *c*/CL complex peroxidase activity is a promising field of investigation for the development of new anti-apoptotic drugs [185,186,187,188].

### 6.2. Cytoglobin

Cygb is a recently discovered protein that is a member of the vertebrate globin family. All globins, namely Hb, Mb, Cygb (MW ~ 21 kD), and neuroglobin (Ngb, MW ~ 17 kD), have a high α–helice globular structure, heme group in the active site, and similar functions: oxygen transport and participation in NO metabolism [62,143,144,145]. Cygb and Ngb have been reported to protect against cell dysfunctions under hypoxia and oxidative stress, which is not the case for Hb and Mb. Ngb is predominantly expressed in the brain. Cygb was discovered in 2001 in hepatic stellate cells [189]. Further studies revealed that the protein is expressed ubiquitously in all tissues and has a number of specific functions, e.g., collagen synthesis, antifibrotic activity, and suppression of cell oncogenic transformation [190]. The low amount of Cygb in cells suggests the probable signaling activity of the protein [71,72].

Unlike Hb and Mb, the heme iron of Cygb and Ngb has a sixth coordination bond [73,143]. The ferrous forms of the proteins reversibly bind oxygen in competition with the distal His81 ligand. Cygb, but not Ngb, was demonstrated to have substantial peroxidase activity [62,189].

The protein heme group of Cygb is sensitive to oxidation of Cys38 and Cys83 which are exposed to the protein surface. The diatomic ligand binding to the protein heme and its nitrite reductase activity is controlled by formation of the disulfide bridge [144,191,192]. Under an oxidative environment, the heme group is transformed into ferric state and stabilized in penta-coordinated state upon oxidation of Cys residues to an internal disulfide. This form of enzyme possesses peroxidase activity (Table 2) [143].

The protein with disulfide bridge has a low temperature EPR signal at g ~ 6 [73,76]. The peroxidase mechanism of Cygb is similar to that of the pseudo-peroxidases discussed above. Interaction of the Cygb heme with H_2_O_2_ results in formation of Fe(IV) oxoferryl π cation, which is converted to Fe(IV) oxoferryl and protein-derived (tyrosyl) radical detected by LT EPR and by spin trapping [76].

The protein with disulfide bridge has a low temperature EPR signal at g ~ 6 [73,76]. The peroxidase mechanism of Cygb is similar to that of the pseudo-peroxidases discussed above. Interaction of the Cygb heme with H_2_O_2_ results in formation of Fe(IV) oxoferryl π cation, which is converted to Fe(IV) oxoferryl and protein-derived (tyrosyl) radical detected by LT EPR and by spin trapping [76].

Reeder et al. demonstrated that the ferric form of Cygb lost its distal His81 ligand after binding of oleic acid or CL [71]. Cygb is potent lipid oxidant. It oxidizes lipids five-fold faster than Mb. However, due to the low concentration of Cygb in cells and the protein degradation under strong oxidative conditions [76], Cygb-induced lipid oxidation is unlikely to cause extensive cellular damage [71,143]. The authors proposed that the physiological function of Cygb is to generate cell signaling lipid molecules under an oxidative environment.

Fatty acid binding is dependent on the redox status of Cys38/Cys83 and occurs only in the Cygb with intramolecularly cross-linked disulfide and already pre-existing significant peroxidase activity of protein [71]. Fatty acids may act only as a co-activator of the peroxidase function of Cygb.

Searching for physiologically relevant lipid regulators of Cygb, Tejero et al. reported that highly negatively charged anionic phospholipids—particularly phosphatidylinositolphosphates (PIP3 and PIP2)—affect the structural organization of the protein and modulate its iron state [72]. Binding of anionic lipids to ferric Cygb resulted in displacement of His81 by a water molecule. Thus, redox catalytic activity can be conferred on Cygb both conjointly and/or independently of cysteine oxidation. The increase in Cygb peroxidase activity correlated with the negative charges of the phospholipids: PIP3(−4) > PIP2(−3) > TOCL(−2) > DOPA(−1). Essentially, the anionic phospholipids induce the peroxidase activity of Cygb at physiologically relevant concentrations. Cygb’s peroxidase activity can be utilized for the peroxidation of anionic phospholipids yielding mono-oxygenated molecular species [72].

The large increase in Cygb peroxidase activity by PIP2 and PIP3 poses new alternatives for the physiological roles of Cygb [72]. Although PIP2 and PIP3 constitute less than 1% of the total membrane lipids, they are components of signaling pathways regulating cell proliferation and survival [193]. Upon oxidative stress, the increase in the amount of PIP3 and the PIP3/PIP2 ratio correlates with Cygb up-regulation. Thus, possible signaling through a Cygb/PIP3 interaction would be amplified under the stress conditions pathway through lipid peroxidation.

Double regulation of Cygb peroxidase activity by the redox state of SH groups and lipid binding can provide fine turning of the synthesis of signaling lipid molecules.

## 7. Conclusions

Peroxidases form a network in the body that may exert both beneficial and adverse effects. Peroxidases have physiological functions that substantially contribute to immunity and to a number of metabolic and signaling processes. However, under certain circumstances, they exert deleterious effects, such as damage to macromolecues in plasma, the activation of cell death, etc. There are special mechanisms to minimize adverse effects of peroxidases (Table 3). The major of them are specific active site structures and/or different types of regulating molecules. Apart from small molecules like NO or CO that interact with the heme iron, special proteins or lipids can bind to hemoproteins and regulate their peroxidase activity. These mechanisms may be a basis for pharmacological strategies aiming to increase beneficial and to minimize deleterious effects of peroxidases.

## Figures and Tables

**Table 1:** Second order rate constants and oxidants produced in the reaction of different forms of iron with H_2_O_2_. Standard reduction potentials (*E_o_*) are presented for oxidative couples: HOX/X-; ^●^NO_2_/NO_2_^−^; phenoxyl radical (Ph-O^●^)/phenolic compound (Ph-OH); HO^●^/H_2_O; HOO^●^/H_2_O_2_.

Form of Iron	k, M^−1^ s^−1^	Reaction Products	E_o_, V pH 7	References
* free ferrous ion, Fe^2+^	76	hydroxyl radical, HO^●^	2.31	[13,14]
		lipid alkoxyl radical, LO^●^	≤1.06	
Lig-Fe^2+^	10^2^–10^4^	HO^●^, LO^●^		[15,16]
free ferric ion, Fe^3+^	0.01–0.02	hydroperoxyl radical, HOO^●^	1.06	[14,17,18]
		lipid peroxyl radical, LOO^●^	≤1.00	
Albumin-heme		phenoxyl radicals, Ph-O^●^	0.4–0.94	[19,20]
		lipid radical, L^●^ (LO^●^, LOO^●^)	0.6	[14]
True peroxidases	(1.1–4.3) × 10^7^	hypochlorous acid, HOCl	1.28	[21,22]
		hypobromous acid, HOBr	1.13	
		hypoiodous acid, HOI	0.78	
		hypothiocyanous acid, HSCN	0.56	
		phenoxyl radicals, Ph-O^●^	0.4–0.94	[20]
		nitrogen dioxide, ^●^NO_2_	1.04	[23]
Hemoglobin	42–43.6	Ph-O^●^, ^●^NO_2_, L^●^, (LO^●^, LOO^●^)		[24,25]
cyt *c*/cardiolipin	~46.4	Ph-O^●^, L^●^, (LO^●^, LOO^●^)		[26,27]
cyt *c*/cardiolipin + FFA-OOH	~5 × (10^3^–10^5^)			[27]

* Fe^2+^ + H_2_O_2_ = Fe^3+^ + ^●^OH + OH^−^—Fenton reaction; Lig—a ligand (ATP, ADP, UTP, [Fe-S] clusters); L—lipid; FFA-OOH—free fatty acid hydroperoxides.

**Table 2:** Peroxidase activity of heme-containing proteins toward different phenolic compounds (25 °C; pH 7.4; H_2_O_2_ = 100 µM) [69,72,75,103,104].

Substrate:Protein	Guaiacol * MW = 127 D Eo ~ 0.95 V	Amplex Red ** MW = 257 D	Etoposide ** MW = 589 D Eo ~ 0.56 V
Hemoglobin	2.26 ± 0.35	10.5 ± 1.8	0.43 ± 0.05
Myoglobin	0.90 ± 0.05	2.2 ± 0.2	0.34 ± 0.02
Cyt *c*/cardiolipin (1:20)	0.15 ± 0.06	1.40 ± 0.15	0.41 ± 0.02
Cytoglobin (S-S)	0.30 ± 0.05	3.2 ± 0.20	0.28 ± 0.04
MPO	660 ± 50	54.5 ± 3.2	0.020 ± 0.005
MPO + phenol	-	355 ± 45	19.5 ± 2.5

* µmol product/min × nmol protein; ** Δmagnitude (A.U.)/min × * nmol protein; Experimental conditions are summarized in Supplementary Materials, Section S2.

**Table 3:** The major mechanisms for regulation of peroxidase activity of hemoproteins.

Hemoproteins	Peroxidase	The Major In Vivo Products	Mechanisms for Activity Regulation	
			Protein Structure	Other Macromolecules
True peroxidases	Myeloperoxidase	HOCl, HOSCN	heme is located at the base of a deep and narrow heme pocket	Ceruloplasmin
	Eosinophil peroxidase	HOBr, HOSCN		Ceruloplasmin
	Lactoperoxidase	HOSCN		
Pseudo- peroxidases	Hemoglobin	L^●^; ^●^NO_2_; Ph-O^●^	Hb(III) loses heme	Haptoglobin, Hemopexin
	Myoglobin	L^●^; ^●^NO_2_; Ph-O^●^		Hemopexin
	Cyt *c*/CL complexes	L^●^; Ph-O^●^	a CL fatty acid occupies cyt *c* active site	
	Cytoglobin	L^●^; Ph-O^●^	oxidation of Cys38 and Cys83 residues to an internal disulfide	Fatty acids and negatively charged lipids

## Supplementary Materials

The following are available online, Section S1: Oxidation-reduction potential, Section S2: Experimental conditions for peroxidase activity measurements, Table S1: Plasma concentrations and second order rate constants for the reactions of selected MPO substrates with Compound I and Compound II, Figure S1: Dityrosine formation induced by Fenton chemistry or catalytically active myoglobin, Figure S2: Low-temperature EPR spectra of high spin ferric heme and protein-based radicals of pseudo-peroxidases, Figure S3: Phenolic compounds—peroxidase substrates, Figure S4: CO binding to cyt *c* depends on cardiolipin concentration.

## Abbreviations

CL	cardiolipin
COX	cyclooxygenase
CP	ceruloplasmin
Cytg	cytoglobin
Cyt *c*	cytochrome *c*
EPO	eosinophil peroxidase
EPR	electron paramagnetic resonance
FFA	free fatty acids
Hb	hemoglobin
Hp	haptoglobin
Hx	hemopexin
L	lipid
LPO	lactoperoxidase
LT	low temperature
Mb	myoglobin
MPO	myeloperoxidase
Ngb	neuroglobin
Ph	phenolic compound
Por	heme porphyrin ring
TLCL	tetralinoleoyl cardiolipin
TOCL	tetraoleoyl cardiolipin
