# Supplementary material for: Peroxidase Activity of Human Hemoproteins: Keeping the Fire under Control
Source: Molecules. 2018 Oct 8;23(10):2561. doi: 10.3390/molecules23102561 (PMC6222727; doi:10.3390/molecules23102561)
Supplement: Supplementary file 1 [file molecules-23-02561-s001.pdf]

Supplementary Materials
for

Peroxidase Activity of Human Hemoproteins:
Keeping the Fire under Control.

Irina I. Vlasova

Federal Research and Clinical Center of Physical-Chemical Medicine, Department of Biophysics,
Malaya Pirogovskaya, 1a, Moscow 119435, Russian Federation
Institute for Regenerative Medicine, Laboratory of Navigational Redox Lipidomics,
Sechenov University, 8-2 Trubetskaya St., Moscow, 119991, Russian Federation

Section S1. Oxidation-reduction potential

The reduction potential is a characteristic of the chemical species to participate in oxidation-reduction reactions. The higher the compound's positive potential, the greater affinity it has for electrons, and thus a higher propensity to steal electrons from another species (this species is oxidized). Conversely, the lower the specie's reduction potential is, the lower its affinity for electrons will be, and thus a higher tendency to donate its electrons and to be a reducing agent. The value of the compound's reduction potential determines its affinity for electrons. A compound with higher reduction potential is able to take electrons from another compound with lower potential if thermodynamically favorable reaction is not constrained by a high activation energy barrier like in the case of H_2O_2 [9]. Free radicals are characterized by high reduction potentials, they are usually oxidizing agents. Antioxidants have low reduction potentials, they have higher tendency to donate electrons and be reducing agents.

Specifically, 'reduction potential of redox couple' should be discussed because reduction potential of a species depends on a product it is converted to in a reaction. Reduction potential of compound I/native MPO (two electron oxidation) is are 1.16 V, but it comprises 1.35V for the pair Compound I/Compound II (one-electron oxidation).

For details:
http://www.molecularhydrogeninstitute.com/oxidation-reduction-potential-orp-explained

Section S2. Experimental conditions for peroxidase activity measurements

Results presented in Table 2

50-100 mM sodium phosphate buffer or 25 mM HEPES-Na, pH 7.4, and 100 µM DTPA, 22-25°C.

1. **Guaiacol** is oxidazed to tetraguiacol which has absorbance spectrum with maximum at 470 nm.

Reaction mixture: 10 mM quaiacol + a protein, the reaction was initiated by 100 µM H_2O_2
Kinetics of guaiacol oxidation was monitored at 470 nM (ε_{470} = 26600 M^{-1}xcm^{-1})
Protein concentrations: 2.5-5 nM MPO; other proteins are at micromolar concentrations (3-5 µM).

2. **Amplex red** is oxidized to resorufin with specific fluorescence at 585 nm (Ex 570 nm)

Reaction mixture: 50 or 100 µM Amplex Red + a protein, the reaction was initiated by 50 µM H_2O_2.
Resorufin fluorescence was measured during 2-3 min after H_2O_2 addition.
Protein concentrations: (20 - 100) nM MPO; (0.5-2) µM other hemoproteins.

3. Oxidation of **etoposide** was detected by measuring the magnitude of EPR spectra of etoposide phenoxyl radicals.

Reaction mixture: (100 or 200) µM etoposide + a protein, kinetics of etoposide radical formation was monitored in 1 min after the addition of 100 µM H_2O_2 to the solution.
Protein concentrations: (200-700) nM MPO (as available); 2.5-5 µM other hemoproteins. EPR settings depended on the magnitude of Etop-O• signal produced in the reaction [72, 75, 103].

Spectrum and kinetics
of etoposide phenoxyl radicals

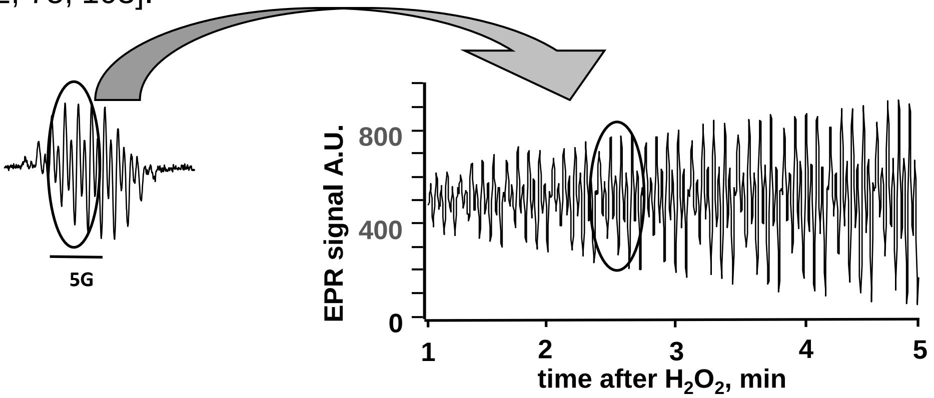

Table S1. Plasma concentrations and second order rate constants for the reactions of selected MPO substrates with Compound I and Compound II at pH 7.0, 15 or 25 °C [51]

Substrate	Concentration in plasma	Rate constant Compound I, M^{-1} s^{-1}	Rate constant Compound II, M^{-1} s^{-1}
Chloride	100-140 mM	2.5×10^4 [28]	-
H_2O_2	< 5 µM [6]	3×10^4 [54]	
Tyrosine	20-80 µM [4]	7.7×10^5 [55]	1.57×10^4 [55]
Ascorbate	20-150 µM [4]	2.3×10^5 [54]	5.0×10^3 [54]
Nitrite	1-50 µM [52]	2.0×10^6 [56]	5.5×10^2 [56]
Urate	200-500 µM [53]	4.6×10^5 [53]	1.7×10^4 [53]

Figure S1. Dityrosine formation induced by Fenton chemistry or catalytically active myoglobin

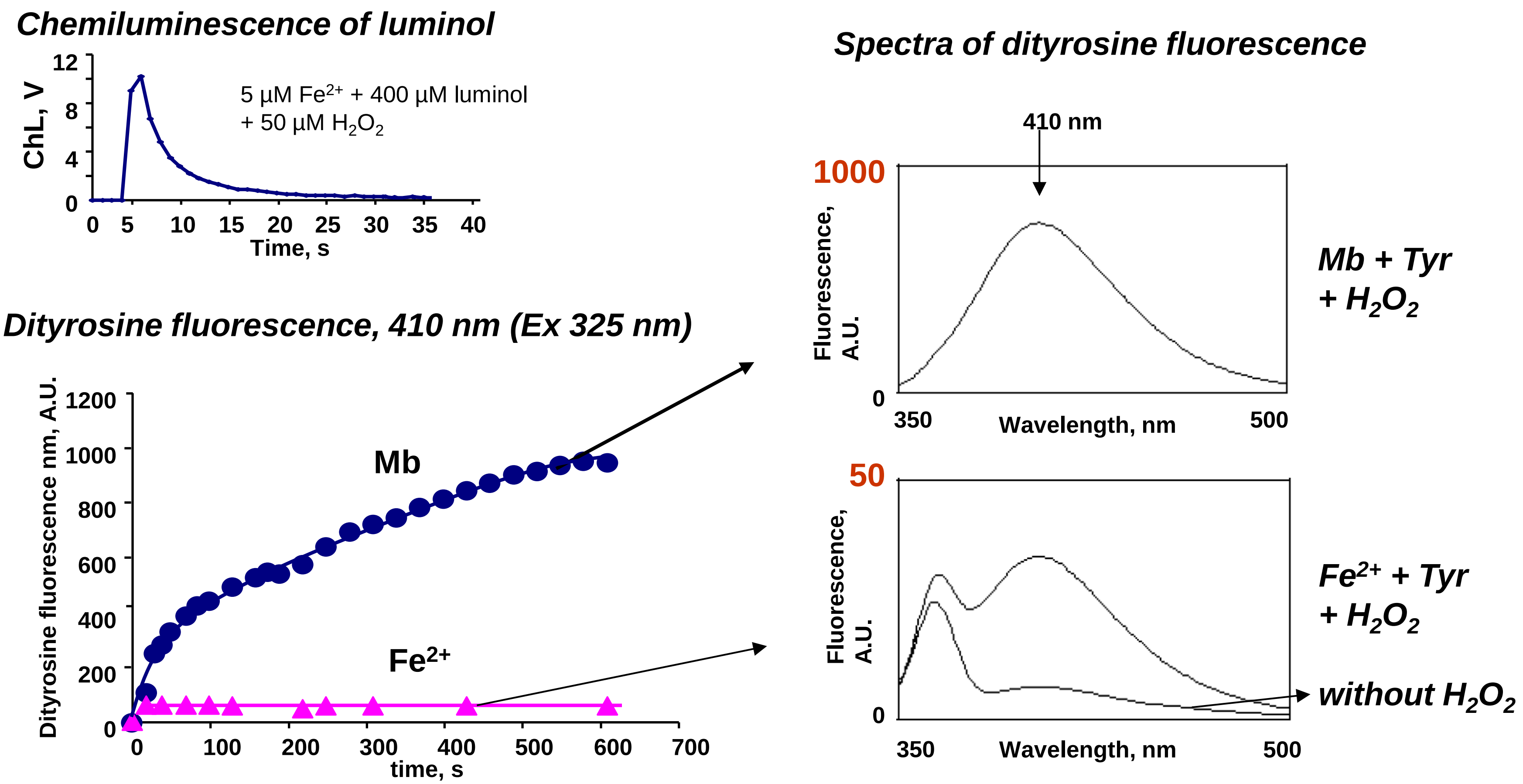

1 µM myoglobin (Mb) or 1.5 µM $FeSO_4$ was added to the solution of 50 µM tyrosine in 10 mM Na-phosphate buffer (pH 7.3), then 50 µM H_2O_2 was added and dityrosine fluorescence was measured at 410 nM (Ex 325 nM). Spectra were recorded at indicated time points.

The high initial rate of dityrosine formation in the solution of Fe^{2+} may be due to iron ligation by phosphate molecules [16].

The amount of dityrosines produced by (Fe^{2+} + H_2O_2) does not exceed 5% of that produced by (Mb + H_2O_2) [65-67].

Oxidation of luminol evidences HO• radical formation in the solution of (Fe^{2+} + H_2O_2).

Figure S2. Low-temperature EPR spectra of high spin ferric heme and protein-based radicals of pseudo-peroxidases

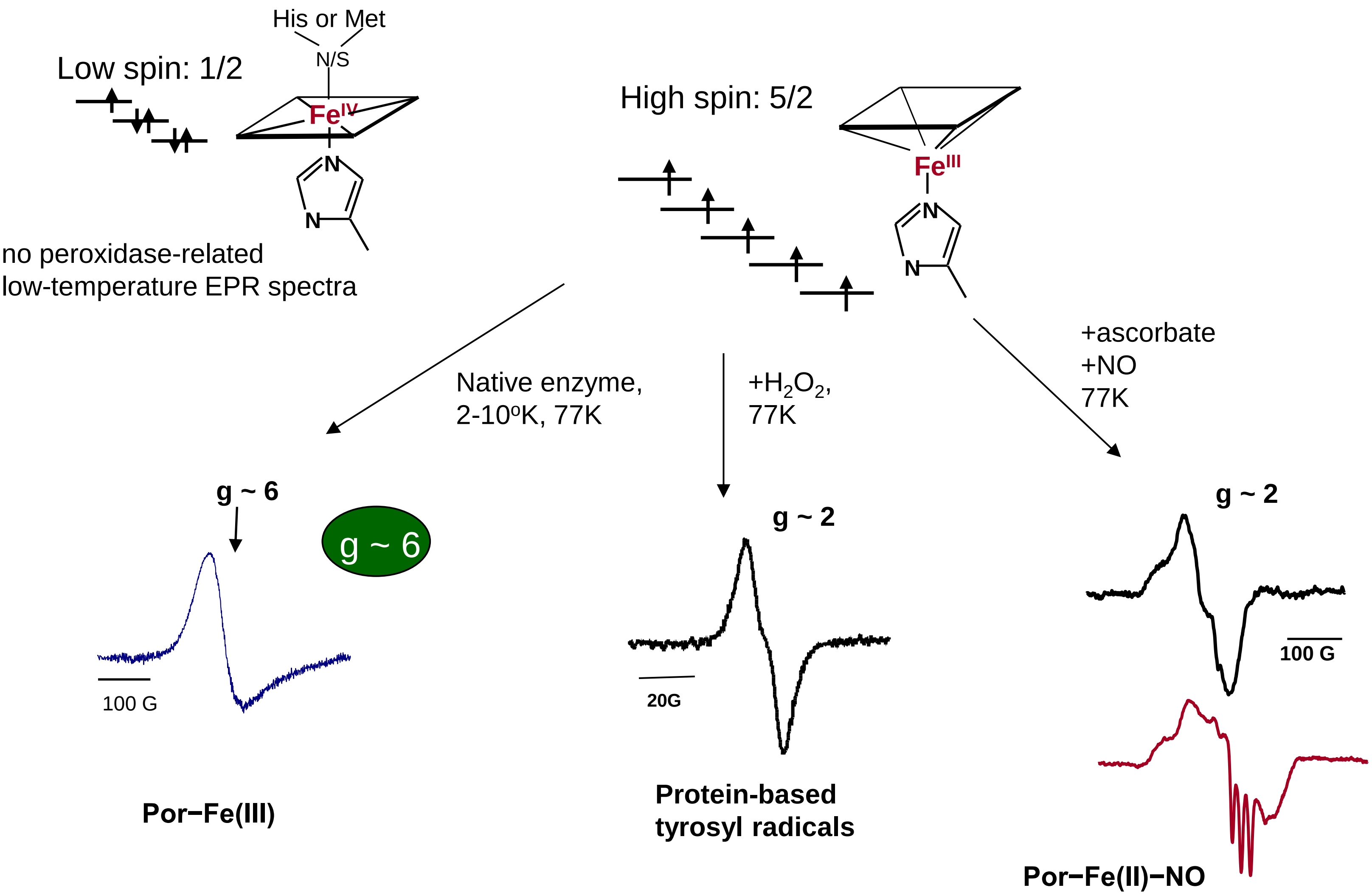

A g-value is (spectrometer frequency/resonance field position)

Figure S3. Phenolic compounds – peroxidase substrates

Guaiacol

MW=127

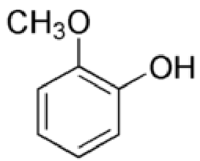

Amplex Red

MW=257

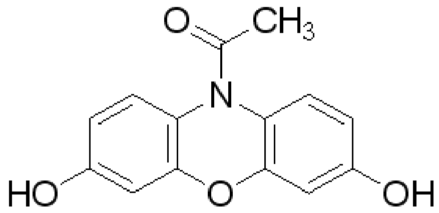

Etoposide

MW=589

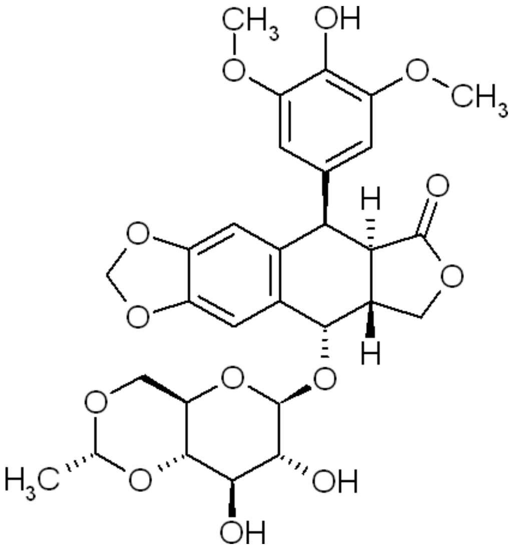

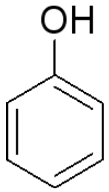

phenol

MW=94

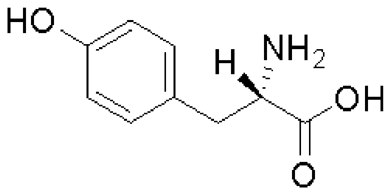

L-tyrosine

MW=121

Figure S4. CO binding to cyt c depends on cardiolipin concentration.

Optical absorbance spectra of cyt c/TOCL complexes in the presence of CO as a function of TOCL concentration

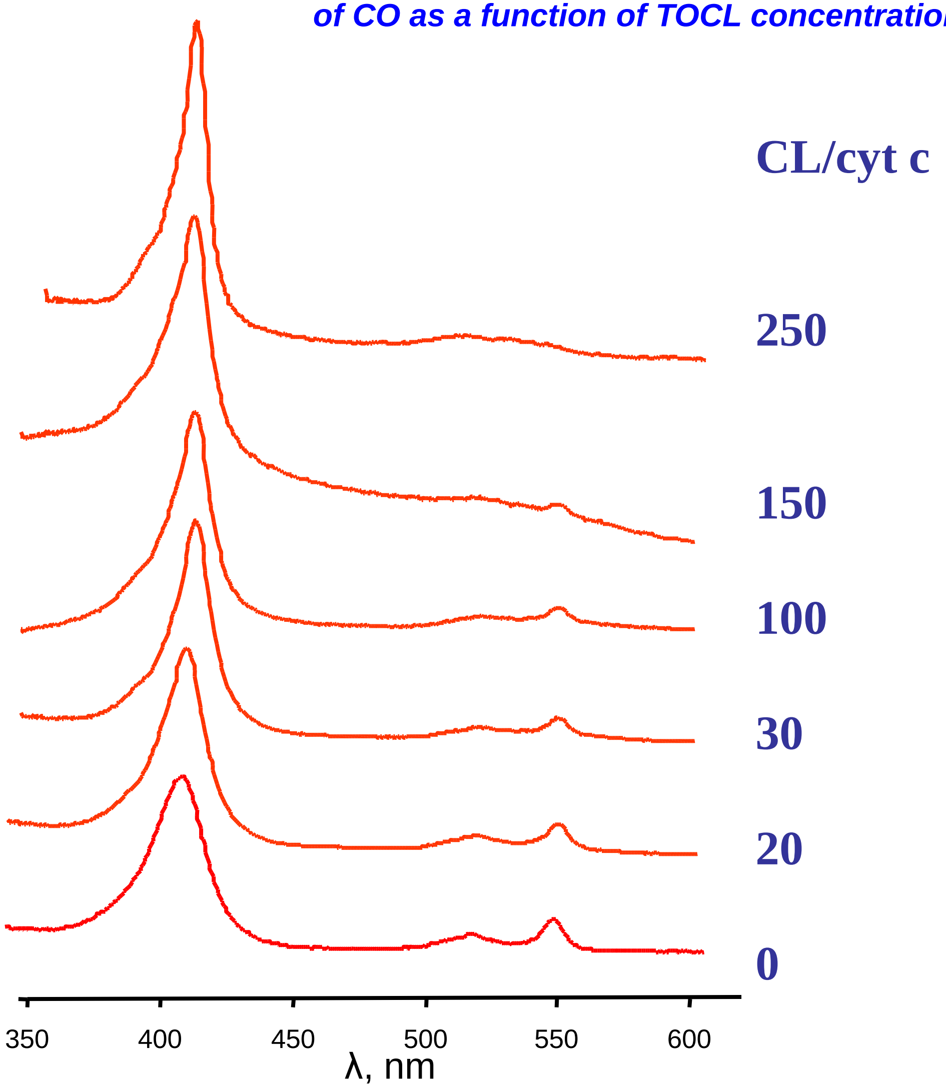

For reduction of cyt *c* to its ferrous form, 10mM ascorbate was added to the solution of cyt *c* (0.5 mM) in 25 mM HEPES-Na buffer, pH 7.4, 100 µM DTPA and incubated for 20 min. The solution was then passed through Sephadex G-25 column (HiTrap Desalting, 1.6 2.5 cm) equilibrated with the same buffer.

.

Cyt *c*(2+) (2 µM) was incubated with TOCL/DOPC liposomes for 15 min under N2 blowing in 25 mM HEPES-Na, pH 7.4 (the buffer was preliminary bubbled with N2), then CO blowdown was added and incubation was continued for 1 h more. Spectra of cyt *c* were recorded using tightly closed cuvette.

TOCL - 1,1’2,2’-tetraoleoyl cardiolipin
DOPC - dioleoyl-L-α-phosphatidylcholine
